# Pravastatin Improves Glucose Regulation and Biocompatibility of Agarose Encapsulated Porcine Islets following Transplantation into Pancreatectomized Dogs

**DOI:** 10.1155/2014/405362

**Published:** 2014-05-19

**Authors:** Lawrence S. Gazda, Horatiu V. Vinerean, Melissa A. Laramore, Richard D. Hall, Joseph W. Carraway, Barry H. Smith

**Affiliations:** ^1^The Rogosin Institute-Xenia Division, 740 Birch Road, Xenia, OH 45385, USA; ^2^The Rogosin Institute, New York, NY 10021, USA; ^3^Florida International University, Miami, FL 33199, USA; ^4^Bob Evans Farms, Inc., New Albany, OH 43054, USA; ^5^NAMSA, Northwood, OH 43619, USA; ^6^NewYork-Presbyterian Hospital and Weill Medical College of Cornell University, New York, NY 10021, USA

## Abstract

The encapsulation of porcine islets is an attractive methodology for the treatment of Type I diabetes. In the current study, the use of pravastatin as a mild anti-inflammatory agent was investigated in pancreatectomized diabetic canines transplanted with porcine islets encapsulated in agarose-agarose macrobeads and given 80 mg/day of pravastatin (*n* = 3) while control animals did not receive pravastatin (*n* = 3). Control animals reached preimplant insulin requirements on days 18, 19, and 32. Pravastatin-treated animals reached preimplant insulin requirements on days 22, 27, and 50. Two animals from each group received a second macrobead implant: control animals remained insulin-free for 15 and 21 days (AUC = 3003 and 5078 mg/dL/24 hr days 1 to 15) and reached preimplant insulin requirements on days 62 and 131. Pravastatin treated animals remained insulin-free for 21 and 34 days (AUC = 1559 and 1903 mg/dL/24 hr days 1 to 15) and reached preimplant insulin requirements on days 38 and 192. Total incidence (83.3% versus 64.3%) and total severity (22.7 versus 18.3) of inflammation on tissue surfaces were higher in the control group at necropsy. These findings support pravastatin therapy in conjunction with the transplantation of encapsulated xenogeneic islets for the treatment of diabetes mellitus.

## 1. Introduction

Type 1 diabetic patients face a lifetime of daily insulin injections due to destruction of the insulin-producing islets of Langerhans within the pancreas. Alternatively, whole pancreas or the islets alone, after isolation from the pancreas, can be transplanted to the patient. Transplantation of either the pancreas or the islets, however, requires a lifetime of immunosuppressive therapy. One possible solution to avoid lifelong immunosuppression is to encapsulate the islets within a semipermeable membrane. The rationale behind this strategy is to protect the islets from destruction by immune cells and immune mediators such as cytokines. The porous membrane should allow the diffusion of insulin and other peptides out of the encapsulation device while still permitting nutrient exchange to support the islet cells.

Following the seminal studies of Lim and Sun [1] in which encapsulated rat islets were shown to restore normoglycemia in diabetic rats, many groups have attempted islet grafting using large animal models as a prelude to clinical trials. Warnock and Rajotte demonstrated the applicability of nonencapsulated islet transplantation in dogs with long-term function of islets autografted to either the spleen or liver [2]. Allogeneic islet transplantation within intravascular devices has also been successful. Sullivan et al. reported 6 of 10 pancreatectomized dogs without exogenous insulin for up to 5 months following the grafting of such devices seeded with allogeneic islets [3]. Brunetti et al. used an intravascular device to at least partially reverse hyperglycemia in spontaneously diabetic dogs grafted with xenogeneic porcine islets [4]. A similar intravascular device study in pancreatectomized dogs that received porcine islets was reported by Maki et al. in which 7 of 17 dogs were maintained on reduced insulin requirements for 53–114 days while 2 animals received reduced insulin for 218 and 263 days [5]. Thrombosis of the vascular shunt, however, remains an ongoing concern for such devices.

To avoid such complications associated with intravascular devices and to limit immunosuppression, many researchers have turned to transplanting encapsulated islets into the peritoneal cavity as free-floating diffusion structures. Soon-Shiong et al. demonstrated 63–172 days of insulin-independence with allogeneic islets microencapsulated in alginate and transplanted into the peritoneal cavity of spontaneously diabetic dogs receiving subtherapeutic doses of cyclosporine [6]. Similarly, reduced insulin requirements and/or insulin independence was reported by Lanza et al. using XM-50 tubular membranes in the absence of immunosuppression [7]. This group later reported insulin-independence for 60 to >175 days in 4 spontaneously diabetic dogs using alginate encapsulated canine islets on low-dose cyclosporine therapy [8]. More recently, Wang et al. were able to discontinue exogenous insulin therapy in 8 of 9 pancreatectomized canines for 64–106 days and in one animal for 214 days using allogeneic islets encapsulated in a triple membrane alginate-based bead without any immunosuppression or anti-inflammatory treatment [9]. Kin et al. demonstrated insulin independence for 6–119 days in pancreatectomized dogs receiving xenogeneic porcine islets encapsulated in agarose and polystyrene sulfonic acid [10]. A bioartificial endocrine pancreas consisting of porcine islets contained in an agarose matrix within semipermeable membranes was also shown to reduce insulin requirements in pancreatectomized dogs for up to 17 weeks [11].

We have reported on the ability of porcine islets encapsulated in a double layer of agarose to eliminate insulin requirements in 12 of 12 spontaneously diabetic BB rats for more than 200 days [12]. In these studies, no immunosuppression was required and the rats remained healthy throughout the study. With a view to clinical trials, we set out to determine the efficacy of agarose-agarose porcine islet macrobead implantation in a large animal model of diabetes mellitus. In the current study, we demonstrate improved functionality and biocompatibility of porcine islet macrobeads in pancreatectomized dogs and administered the HMG-CoA reductase inhibitor pravastatin for its mild anti-inflammatory properties [13] as compared to diabetic dogs that did not receive pravastatin.

## 2. Materials and Methods

### 2.1. Animals

A total of six male devocalized Beagle dogs (Ridgelan Farms, Inc.) were received at approximately 25 weeks of age. The dogs were individually housed in stainless steel cages, 32′′W × 42′′L × 32′′H, with rubbercoated mesh flooring and a stainless steel platform, 32′′ × 40′′. Science Diet Growth (Hill's, 6730) was given to the animals twice daily. Clean municipal water was provided* ad libitum*. Viokase (Henry Schein, 9758341) powder was added to food at a dose of 1 tsp/meal following pancreatectomy. The temperature of the room was maintained within 18–28°C with relative humidity of 30–70%. A 12 hour light cycle was maintained throughout the study with lights on at 0700 hours. All study protocol procedures were approved by The Rogosin Institute Animal Care and Use Committee (IACUC). The Rogosin Institute-Xenia Division animal facility holds Full Accreditation status awarded by the Association for Assessment and Accreditation of Laboratory Animal Care, International (AAALAC, Int.).

### 2.2. Isolation of Porcine Islets and Production of Porcine Islet Macrobeads

Donor islets were prepared from Newsham sows over two years of age and with multiple parities. After electrical stun and exsanguination (Bob Evans Farms, Xenia, OH), pancreata were retrieved and transported to the islet isolation laboratory in cold Hank's Balanced Salt Solution (HBSS; Mediatech, 21-020) on ice. Warm ischemia times averaged 14.43 minutes while cold ischemia times ranged from 30 minutes to one hour. Islet isolation was carried out as previously described [14]. Briefly, after trimming the gland of fat and connective tissue, the main pancreatic duct was cannulated and injected with HBSS containing collagenase V (Sigma Aldrich, C9263) at a concentration of 1.8 g/L, and protamine (Sigma Aldrich, P4005) at a concentration of 0.06 g/L. Four times the gram weight of the pancreas (in mL) was perfused through the pancreatic duct at a rate of 150 mL/min at 18°C. Islets were purified on discontinuous Eurocollins (Mediatech, 99-408)—Ficoll (Sigma Aldrich, F9378) gradients of densities 1.105 g/cm^3^, 1.095 g/cm^3^, and 1.055 g/cm^3^ in 50 mL polystyrene conical tubes. The tubes were centrifuged at 2000 RPM, and islet-containing layers were manually collected. 500 islet equivalents were encapsulated in each agarose-agarose macrobead as previously described [14]. Islet macrobeads were maintained in RPMI containing 2.5% porcine serum (Mediatech, 35-041) and 1% antibiotic/antimycotic (A/A; Gibco Life Technologies, 15240) in an atmosphere of 4–6% CO_2_ and air at 36–38°C. Macrobeads were examined for uniformity, collected the day prior to implant and were aliquoted to 175 mL conical tubes. A maximum of 400 macrobeads per tube were stored overnight at room temperature (19–27°C) in RPMI (Gibco Life Technologies, 22400) containing 1% A/A.

### 2.3. Microbiology Screening of Macrobeads for Implantation

Four weeks prior to islet macrobead implantation, representative samples of macrobeads and culture media were sent to LabCorp (Columbus, OH) for bacterial and fungal testing (Bioburden Process). The macrobeads were aseptically crushed (culture media tested as supplied) and supernatant cultured on Sabouraud and Mycosel media (28 days) and blood and MacConkey Agar (48 hr).

### 2.4. Surgical Procedures

Animals were sedated with acepromazine at a dose of 0.05 mg/kg IM (Henry Schein, 356-7290) and anesthetized with ketamine at a dose of 5 mg/kg (Fort Dodge Animal Health, 9952949), diazepam at a dose of 1 mg/kg (Butler, GNR04054), and isoflurane gas at 1–3% (Henry Schein, 982-2413). During surgery all animals received buprenorphine (Butler, GNR02300) at doses of 0.005–0.020 mg/kg IM during surgery and every 8–12 hours postsurgery, continuing for 2-3 days; and cefotaxime (claforan antibiotic; Henry Schein, 852-1633) at a dose of 20 mg/kg IV during and 8 hours postsurgery. At implant, the abdominal, subcutaneous, and cutaneous incisions were closed with 3–0 vicryl (Ethicon, J333), 3–0 vicryl, and 3–0 nylon (Ethicon, J669) sutures, respectively. At pancreatectomy, the abdominal, subcutaneous, and cutaneous incisions were closed with 2–0 vicryl (Ethicon, J332), 4–0 polymend (Veterinary Products Laboratories, V-397-1), and 3–0 nylon (Ethicon, J669H) sutures, respectively.

Seven weeks following arrival, all animals, with the exception of VPL-0, underwent pancreatectomy for the induction of insulin-dependent diabetes. VPL-0 replaced an unsuitable study animal during week three and was therefore pancreatectomized at four weeks after arrival. The pancreas was exposed by a peritoneal midline incision, approximately 5–8 cm long. The pancreas was removed with blunt dissection, and the site of pancreatectomy was photographed. At the time of pancreatectomy, the majority of the omentum was also removed. Following induction, animals were maintained on Humulin 70/30 insulin (Eli Lilly, 029363) at doses to attempt morning (0900 hr) and evening (1800 hr) normoglycemia. Exogenous insulin therapy was provided following pancreatectomy and was also resumed postmacrobead implantation when a consistent increase in blood glucose above normoglycemia was observed for at least 3 days.

### 2.5. Porcine Islet Macrobead Implantations

Porcine islet macrobeads were implanted into the peritoneal cavities of all six animals 26 weeks following pancreatectomy, at a dose of 1.85 × total daily exogenous insulin requirements. Fifteen weeks following the initial macrobead implant, a second implant of porcine islet macrobeads was performed in four of the six animals at a dose of 2.71 × total daily exogenous insulin requirements. Pravastatin (80 mg/day, Teva Pharmaceuticals) was administered beginning on the day of islet macrobead implantation and was continued, daily, throughout the course of the study.

Because the in vivo environment of the dog abdomen would likely impair insulin production from implanted macrobeads as compared to the carefully controlled in vitro culture environment, we opted to implant dogs with enough macrobeads to provide 1.85 times (1.85 × dose for the 1st implant) or 2.71 times (2.71 × dose for the 2nd implant) the amount of each dog's daily exogenous insulin requirement. Once a week prior to macrobead implantation, insulin production during a 24-hour period of in vitro culture was determined for each batch of islet macrobeads. For implant purposes, the average daily insulin secretion per macrobead in the four weeks prior to each implant was considered (Table 1). The daily exogenous insulin requirements of each macrobead implanted dog were multiplied by 1.85 (first implant) or 2.71 (second implant) and enough macrobeads were implanted to provide the desired dose of insulin. Since insulin production varies with every batch of islet macrobeads, each preparation of islet macrobeads was evenly divided amongst the different recipient dogs such that each animal received a proportion of each batch of macrobeads based on exogenous insulin requirements (Table 1). For the first implant, each dog received 737–1081 porcine islet macrobeads with a total macrobead weight of 195.2–285.9 g. For the second macrobead implant each dog received 923–1565 porcine islet macrobeads weighing 244.7–415.0 g. Average macrobead age (time postisolation and encapsulation) was 12 weeks for all dogs at the time of the first implant and 10 weeks for the second implant.

A peritoneal midline incision was made and the site of pancreatectomy was examined for the presence of any residual pancreatic tissue just prior to placing the macrobeads into the peritoneal cavity. Immediately prior to implant, macrobeads were washed three times with RPMI containing 1% A/A and were gently placed into the peritoneal cavity by use of a sterile plastic spoon. As needed, animals were manually fed Hill's Prescription Diet A/D (5670), Hill's canned Science Diet Growth (6680), and/or Nutri-Cal (Evsco Pharmaceuticals, 01311) to maintain normoglycemia during the first 24–48 hr postimplant.

### 2.6. Clinical Observations

Individual animal medical observations were recorded daily throughout the study. Blood glucose was initially determined using Accu-chek advantage blood glucose monitor and Chemstrips (Roche Diagnostics) and had a maximum value of 500 mg/dL. Later in the study Accu-Chek Simplicity BG monitor and Chemstrips (Roche Diagnostics) were used and had a maximum value of 600 mg/dL. Intravenous glucose tolerance tests (IVGTTs; 50% dextrose in sterile water, 1.0 g dextrose/kg) were also performed throughout the study.

### 2.7. Porcine Insulin and C-Peptide Assays

Standard radioimmunoassays, from Linco Research, Inc., were used for the detection of porcine insulin (PI-12K, sensitivity of 2 *μ*U/mL) and porcine C-peptide (PCP-22K, sensitivity of 0.1 ng/mL). Assays were run with samples in duplicate and reference, standards, and controls in triplicate according to the manufacturer's instructions.

### 2.8. Necropsy

Complete necropsies were performed 29 weeks following a second implant of porcine islet macrobeads. Following anesthesia and collection of blood and exsanguinations, macrobeads were randomly selected for histopathology. Macroscopic observations were noted and major organ systems and tissues were photographed. Weights of major organs were recorded. Tissue samples, including porcine islet macrobeads, were either aliquoted to frozen storage (−70°C) or fixed in 10% neutral buffered formalin and sent to Pathology Associates International (PAI, A Charles River Company) for histopathology.

### 2.9. Histopathology

At the time of pancreatectomy, both pancreas and omentum were fixed in 10% neutral buffered formalin for 24 hr at which time tissues were washed and stored in 70% ETOH. At necropsy, the following tissues were collected, fixed as above, and sent for histopathology: heart, spleen, liver, kidneys, brain, testes, duodenum, jejunum, ileum, mesentery, adrenal glands, stomach, lungs, diaphragm, abdominal musculature, bone (sternum), spinal cord, sciatic nerve, epididymis, eyes, submandibular lymph nodes, popliteal lymph nodes, and mesenteric lymph nodes. Tissues were embedded in paraffin, and 5 *μ*m sections were stained with haematoxylin and eosin (H&E). Samples were analyzed by a board-certified pathologist and the macroscopic and histopathological findings were documented. All histopathology was performed according to standard operating procedures of PAI.

## 3. Results

### 3.1. Insulin Requirements and Individual Blood Glucose

**Figure 1:**
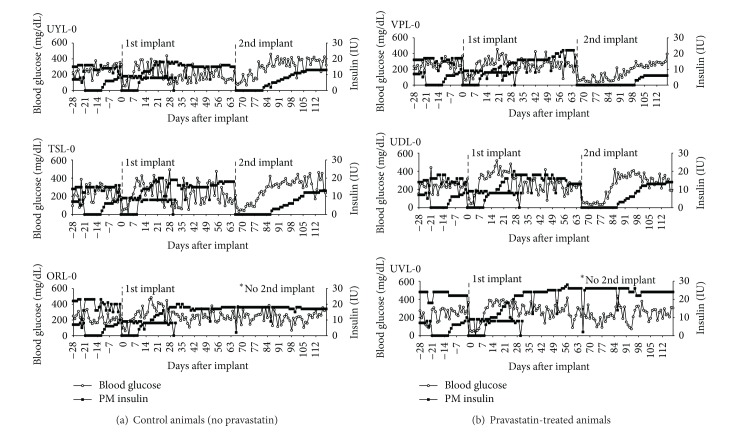
Blood glucose levels of study animals are shown as an average of daily AM and PM blood glucose levels (open circles). Exogenous insulin totals are graphed as a sum of daily AM and PM insulin units received (closed squares). Macrobead recipients that did not receive pravastatin (control animals) are shown in panel (a) (UYL-0, TSL-0, and ORL-0). Macrobead recipients that received daily pravastatin (80 mg/day) are shown in panel (b) (VPL-0, UDL-0, and UVL-0). ORL-0 and UVL-0 did not receive a second porcine islet macrobead implant.

Following the first macrobead implantation, all animals remained insulin-free for a period of nine days despite the implantation of a suboptimal critical mass of islet tissue (Figure 1). On the tenth day following macrobead implantation, all six animals were started on exogenous insulin therapy in response to rising blood glucose levels. Insulin requirements gradually increased to preimplant levels over the following 8–40 days. Non-pravastatin-treated animals reached preimplant insulin requirements on days 18, 19, and 32 while pravastatin-treated animals reached preimplant insulin requirements on days 22, 27, and 50 following the implant.

Following the second islet macrobead implant, the two non-pravastatin-treated dogs (UYL-0 and TSL-0) began exogenous insulin therapy on days 16 and 21, respectively (Figure 1(a)). Before second implant, insulin requirements for the non-pravastatin-treated dogs were reached on days 62 and 131. The two pravastatin-treated animals (UDL-0 and VPL-0) began insulin therapy on days 21 and 34, respectively. Before second implant, insulin requirements for these dogs were reached on days 38 and 192 (Figure 1(b)). A more gradual return to preimplant insulin requirements was observed for both groups of animals following the second macrobead implant, although a suboptimal dose of islet tissue was again transplanted. Using the total area under the curve with respect to ground [15], the glucose area under the curve (AUC) was calculated for the period of time that all four of these animals remained insulin-free (day 1 to 15 after the second implant). The two pravastatin-treated animals, UDL-0 and VPL-0, had an AUC of 1559 mg/dL/24 hr and 1903 mg/dL/24 hr, respectively. The AUC for the non-pravastatin-treated animals, TSL-0 and UYL-0, was 3003 mg/dL/24 hr and 5078 mg/dL/24 hr, respectively (*P* = 0.15 between groups, two-tailed* t*-test). Importantly, normoglycemia was maintained throughout most of the insulin-free period for both groups of animals (Figure 1).

### 3.2. IVGTT

**Figure 2:**
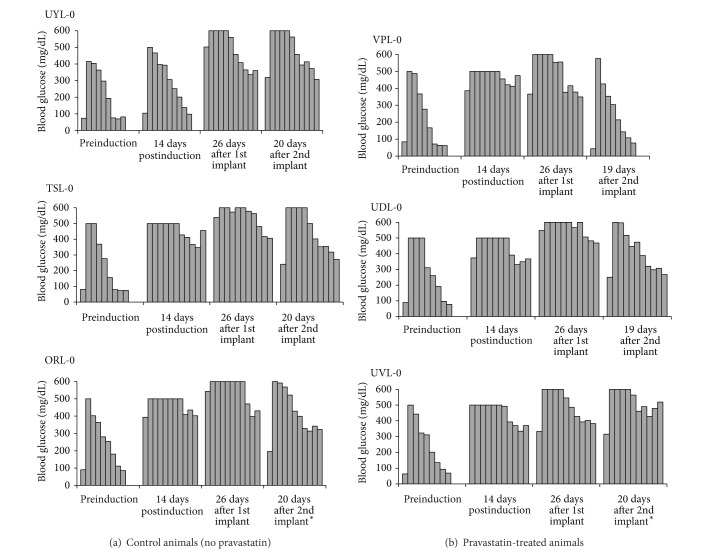
Blood glucose levels during IVGTTs at time = 2, 5, 10, 15, 30, 60, 90, 120, and 240 minutes following dextrose administration. Control animals (did not receive pravastatin) are shown in panel (a) (UYL-0, TSL-0, ORL-0). Macrobead recipients that received pravastatin are shown in panel (b) (VPL-0, UDL-0, UVL-0). ORL-0 and UVL-0 did not receive a second porcine islet macrobead implant.

Individual blood glucose levels taken during IVGTTs are presented in Figure 2. Prior to the surgical induction of diabetes, all animals reached a peak glucose level by 2 minutes after the infusion of dextrose which then returned to baseline levels by 2 hours. Following pancreatectomy, baseline hyperglycemia was noted in five of the six study animals. One animal, UYL-0, demonstrated a similar response as to preinduction dextrose challenge (Figure 2(a)). All animals were receiving insulin therapy at this time.

Nineteen to twenty days following the second macrobead implant, all study animals were again challenged. Both non-pravastatin-treated animals returned to prechallenge blood glucose levels, although hyperglycemia was still present four hours after the procedure. One of these animals, UYL-0, began insulin therapy five days prior, making interpretation difficult. The other non-pravastatin-treated dog, TSL-0, was not receiving exogenous insulin and demonstrated some ability to respond to a glucose challenge and return to a prechallenge blood glucose level. Although neither of the two animals on pravastatin received exogenous insulin therapy at the time of the IVGTT following the second implant, both animals demonstrated a significant glucose response as prechallenge levels were reestablished. One animal, VPL-0, demonstrated the notable ability to return to prechallenge normoglycemia, although this response was slower than preinduction (2 hours versus 30 minutes, Figure 2(b)). The two pravastatin-treated animals, UDL-0 and VPL-0, had an AUC of 47,541 mg/dL/min and 21,462 mg/dL/min, respectively. The AUC for the non-pravastatin-treated animals, TSL-0 and UYL-0, was 52,321 mg/dL/min and 57,589 mg/dL/min, respectively (*P* = 0.264, two-tailed* t*-test). Because VPL-0 was able to return to prechallenge normoglycemia by 120 minutes postdextrose administration (and therefore blood glucose measurements for VPL-0 were not taken beyond that point), only this time period (0 minutes to 120 minutes) was considered for the glucose AUC calculations for all animals.

### 3.3. Body Weights

**Figure 3:**
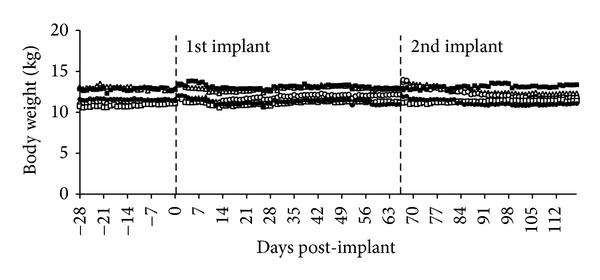
Individual body weights of study animals. Open markers indicate body weights of animals that did not receive pravastatin (UYL-0: open triangle, TSL-0: open circle, ORL-0: open square). Shaded markers indicate body weights of animals that did receive pravastatin (VPL-0: triangle, UDL-0: circle, UVL-0: square). ORL-0 and UVL-0 did not receive the second macrobead implant.

All animals lost body weight immediately after pancreatectomy (Figure 3). Over the next several months, all animals regained weight and surpassed preinduction weights. At the time of the first macrobead implant an average weight gain of 1.76 kg was observed. A slight increase in body weight is also observed in the immediate period following both macrobead implantations, as a result of macrobead weight and fluid therapy associated with the surgical procedures. No significant differences in body weights were observed between pravastatin and non-pravastatin-treated animals.

### 3.4. Blood Chemistry and Complete Blood Count

Complete blood biochemical tabulated profiles are provided as Supplementary Figure S1 in Supplementary Material available online at http://dx.doi.org/10.1155/2014/405362. Average glycosylated hemoglobin was high throughout the study for both groups of dogs following pancreatectomy. Average fructosamine levels, however, were in the normal range throughout the study for both groups of animals. There were no significant differences in biochemical indices between groups. Immunoglobulin levels were variably expressed throughout the study with no apparent trend to associate the findings with any particular procedure or condition. However, IgG and IgM were elevated throughout the study for both groups of dogs following pancreatectomy and IgA appeared to increase approximately 2–4-fold after postimplant day 82 and remained high through the rest of the study. Complete blood counts were determined throughout the study (Supplementary Figure S2).

### 3.5. Necropsy

**Figure 4:**
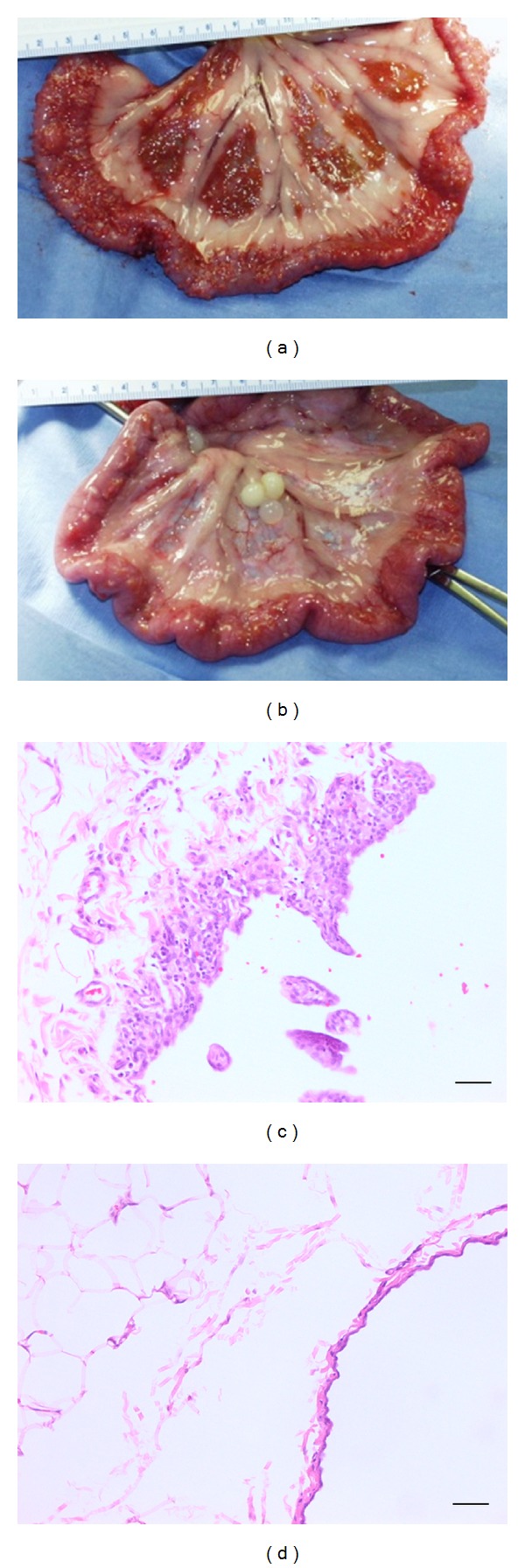
Gross and microscopic images of the mesentery from two representative study animals. Small intestine and mesentery from a control (nonpravastatin) treated animal (ORL-0; (a)) and pravastatin-treated animal (VPL-0, (b)). Microscopic images of the same tissues from these animals are shown in (c) (ORL-0) and (d) (VPL-0). Original magnification of (c) and (d) was 200x. Scale bar is 200 *μ*m.

Complete necropsies were performed on all study animals 29 weeks following the second macrobead implant. During the exploration of the peritoneal cavity, it was noted that approximately 80–90% of the macrobeads were free-floating with the remainder of the macrobeads primarily engulfed in either residual omentum or mesentery. Moderate to severe coalescing proliferative lesions were noted on the abdominal wall of all animals. These lesions were more pronounced along the ventral aspect and within the central portion of the abdominal wall along the incision site. Both the dorsal and outer quarters (superior and inferior to the central region) of the abdominal wall were less severe in nature. Multifocal, raised, white foci were noted on the splenic capsular surface in five animals, and scattered plaques were limited to the splenic tail region of one dog (TSL-0). Moderate proliferative plaques were routinely noted on the serosal surfaces of the intestines and mesentery (Figure 4). A slight thickening of the kidney capsule along with raised white pinpoint foci was recorded for all six animals. Occasional plaques were noted on the surfaces of the liver of five animals.

Overall, the proliferative lesions appeared to be limited to the serosal surfaces, did not appear to extend into organ parenchymal cells at sectioning, and were not thought to impair organ function. There were no clear macroscopic differences between pravastatin and non-pravastatin-treated animals.

### 3.6. Histopathology

Tissues and macrobeads collected at necropsy were processed and examined by a board-certified pathologist. A few viable islet cells were found in the macrobeads from all three pravastatin-treated dogs while no viable islet cells were found in macrobeads retrieved from non-pravastatin-treated animals. The majority of macrobeads from both groups of dogs contained cellular debris. Some macrobeads were surrounded by a proliferative inflammatory capsule characterized by collagenous tissue, vessels, mesothelium, and a few inflammatory cells. Occasional inflammatory cells were also noted along cracked surfaces within macrobeads.

An inflammatory process characterized by fibrous tissue, vascularization, mesothelium, and some inflammatory cells was the most common finding from peritoneal tissues for both groups of dogs. The inflammation presented as irregular villous-like projections and was classified as mild to moderate in nature (Figure 4). A chronic inflammatory process was also associated with the serosa and subserosa of thoracic tissues from two non-pravastatin-treated dogs (TSL-0 and UYL-0). Thoracic tissues from two pravastatin-treated animals (UVL-0 and UDL-0) were also determined to contain subserosal inflammation. The subcapsular sinus and lymphatics of the sternal lymph node from one dog (TSL-0) were found to contain fragments of an inflammatory tag. Overall, the total incidence (83.3% versus 64.3%) and total severity (22.7 versus 18.3) of inflammation on tissue surfaces were higher in the non-pravastatin-treated group (Table 2).

## 4. Discussion

The results of this study are consistent with the hypothesis that the administration of a mild anti-inflammatory agent and an increase in the critical mass of islet tissue following a second implant promote islet macrobead function. All dogs were insulin-free for nine days following the first macrobead implant, but rapidly returned to preimplant exogenous insulin requirements. All implanted dogs, however, were able to maintain a longer period of insulin independence following a second macrobead implant. In addition to the longer insulin-free period following the second implant, a more gradual increase to preimplant insulin requirements was observed for both groups of dogs. Importantly, this more gradual increase in exogenous insulin was associated with normoglycemia as well as steady or increasing body weights.

The observation that macrobead recipients gradually returned to preimplant insulin requirements is somewhat difficult to interpret: the preimplant insulin requirements may not be a completely accurate guide. That is, imbalances of glucagon secretion from the alpha cells within the islet macrobeads and perhaps other peptides, as well as the physiological changes and stresses induced by surgery and the inflammatory process, may change the postimplant insulin requirements (with or without macrobead implants). In this light, the encapsulated islets may have been subjected to greater insulin production demands than native pancreatic islets.

Also notable is the finding that some animals demonstrated the ability to respond to a glucose challenge following the second macrobead implantation. Particularly significant was the ability of VPL-0 to return to normoglycemia during an IVGTT procedure. Although the attainment of normoglycemia during IVGTT was significantly slower than preinduction (2 hours versus 30 minutes) this result clearly demonstrates the ability of porcine islet macrobeads implanted into the peritoneal cavity to respond to a glucose challenge. The slower response of the peritoneal islet macrobeads to restore normoglycemia is not unexpected. The intravenous injection of glucose would likely reach vascularized native pancreatic islets sooner than encapsulated islets free-floating in the peritoneal fluid. In addition, the slower return to normoglycemia can be at least partially explained by the requirements of glucose and insulin to diffuse into and out of the agarose-agarose macrobead.

Arita et al. previously reported the ability of pravastatin to enable the long-term restoration of normoglycemia following the grafting of a subtherapeutic dose of autologous canine islets [16]. Although the normoglycemic and insulin-free period following macrobead implantation was limited in our study, pravastatin-treated animals demonstrated improved glucose regulation as compared to non-pravastatin-treated animals during the period of insulin independence. A constraint of our study is the small number of animals in each group which limits statistical analysis. Nonetheless the improved glucose regulation for the pravastatin group, as reflected in the AUC analysis for both the daily blood glucose readings and for the IVGTT at the end of the insulin-independent period following the second implant is apparent.

Although the majority of macrobeads were found to be free-floating in the peritoneal cavity during the second implant procedure and during necropsy, some macrobeads were surrounded by a proliferative inflammatory tissue capsule. It is not known why a few macrobeads became enveloped in connective tissue while other beads remained free of fibrotic tissue. The observed inflammation was limited to the serosal surfaces of peritoneal organs, suggesting the macrobeads were well tolerated in the peritoneal cavity of study animals.

There are several mechanisms that could account for the cellular death observed within the macrobeads including nutritional deficiency, soluble immune mediators, and inadequate islet mass. It is well known that the oxygen partial pressure within the peritoneal cavity (40 mmHg) is considerably lower than the 100 mmHg in the arterial circulation. The development of fibrous tissue around the macrobeads would certainly exacerbate any oxygen deficiency. The generalized inflammatory response observed in the peritoneal cavity of implanted dogs would also be expected to result in the production of inflammatory cytokines and oxygen radicals, which have both been shown to be toxic to islets. The small molecular size of inflammatory cytokines and oxygen radicals such as nitric oxide and superoxide would easily penetrate the macrobeads. Despite the transplantation of enough islet macrobeads to provide approximately 2-3 × the estimated exogenous insulin requirement, it is possible that the insulin production during in vitro culture is not reflective of insulin production in vivo, in which case an inadequate islet mass may have been transplanted. Limitations in the ability to administer insulin more than twice a day and in the length of action for Humulin 70/30 could have led to an underestimation of the exogenous insulin requirements and thus exacerbated the possibility of implanting an inadequate islet mass.

An enormously valuable and unique feature of the agarose-agarose islet macrobeads is the ability to withstand prolonged periods of in vitro culture while maintaining cellular viability and appropriate physiological responses. This provides the distinct opportunity to thoroughly test the macrobeads for microbiological safety and hormone production prior to transplantation. Appropriate numbers of islet macrobeads producing a known amount of insulin can thus be selected based upon individual insulin requirements.

The findings from this study support continued investigations into the transplantation of encapsulated xenogeneic islets, concurrent with pravastatin therapy, as an option for the treatment of diabetes mellitus. The period of reduced insulin requirements, especially following the second implant, as well as the fasting and glucose-challenged blood glucose levels demonstrate appropriate physiological islet function within the agarose macrobead. The improved macrobead function following the second implant emphasizes the requirement to achieve a critical mass of islet tissue for optimal islet function. The biocompatibility of the agarose-agarose islet macrobeads and the long-term health of the study animals provide further support for this approach to islet xenotransplantation.

## Supplementary Material

Complete blood biochemical profiles (BBP, including fructosamine and glycosylated hemoglobin) and complete blood counts (CBC) were performed on all study animals prior to induction, three weeks post-induction, and every month thereafter. The tabulated results of this testing is provided for each study animal as Supplementary Figure S1 (BBP) and Figure S2 (CBC).
Supplementary Figure S1: Biochemical indices of individual study animals. UYL-0, TSL-0, and ORL-0 did not receive pravastatin. VPL-0, UDL-0 and UVL-0 received pravastatin treatment. Indices were assessed pre/post-induction, 19-, 53-, and 82-days post-macrobead implant #1, and 11-, 41-, and 187-days post-macrobead implant #2. ORL-0 and UVL-0 did not receive a second macrobead implant.
Supplementary Figure S2: Complete blood count profiles of individual study animals. UYL-0, TSL-0, and ORL-0 did not receive pravastatin. VPL-0, UDL-0 and UVL-0 received pravastatin treatment. CBC profiles were assessed pre/post-induction, 19-, 53-, and 82-days post-macrobead implant #1, and 11-, 41-, and 187-days post-macrobead implant #2. ORL-0 and UVL-0 did not receive a second macrobead implant.

## Figures and Tables

**Table 1:** Individual dog insulin requirements and islet macrobead information at first and second implant.

					Porcine islet macrobead Information			
		Animal ID	Body weight (kg)	Daily insulin requirement (IU)	Daily insulin production (IU/bead)	Number of macrobeads implanted	Total daily insulin (IU) from implanted macrobeads	Weight of implanted macrobeads (kg)
First implant	Control	UYL-0	12.88	15	0.0378	736	27.82	0.195
		TSL-0	11.48	16	0.0378	788	29.79	0.207
		ORL-0	11.11	20	0.0378	981	37.08	0.256
	Pravastatin	VPL-0	11.61	17	0.0378	835	31.56	0.219
		UDL-0	11.39	14	0.0378	786	29.71	0.205
		UVL-0	12.79	22	0.0378	1079	40.79	0.285

Second implant	Control	UYL-0	12.93	15	0.0382	1065	40.68	0.286
		TSL-0	12.20	18	0.0382	1278	48.82	0.343
		ORL-0	11.20	18	—	—	—	—
	Pravastatin	VPL-0	12.11	22	0.0382	1565	59.78	0.415
		UDL-0	10.98	13	0.0382	923	32.26	0.245
		UVL-0	12.93	26	—	—	—	—

**Table 2:** Histopathology scoring.

Tissue		Nonpravastatin Control Animals	Pravastatin- treated animals
Heart	Incidence	1/3 (33%)	1/3 (33%)
	Severity	0.3	0.3

Lung	Incidence	2/3 (67%)	1/3 (33%)
	Severity	1.0	0.3

Diaphragm	Incidence	3/3 (100%)	3/3 (100%)
	Severity	2.3	3.0

Liver	Incidence	2/3 (67%)	1/3 (33%)
	Severity	0.7	0.7

Kidney	Incidence	3/3 (100%)	1/3 (33%)
	Severity	1.7	0.7

Spleen	Incidence	3/3 (100%)	3/3 (100%)
	Severity	2.0	1.7

Adrenal Glands	Incidence	2/3 (67%)	2/3 (67%)
	Severity	0.7	0.7

Stomach	Incidence	3/3 (100%)	2/3 (67%)
	Severity	2.7	1.3

Duodenum	Incidence	3/3 (100%)	3/3 (100%)
	Severity	2.7	2.7

Jejunum	Incidence	3/3 (100%)	2/3 (67%)
	Severity	2.0	1.3

Ileum	Incidence	3/3 (100%)	3/3 (100%)
	Severity	2.7	2.3

Mesentery	Incidence	3/3 (100%)	1/3 (33%)
	Severity	1.3	0.7

Mesenteric Lymph Node	Incidence	2/3 (67%)	1/3 (33%)
	Severity	1.0	0.3

Abdominal Wall	Incidence	2/3 (67%)	3/3 (100%)
	Severity	1.3	2.3

Total	**Incidence**	**35/42 (83.3%)**	**27/42 (64.3%)**
	**Severity**	**22.7**	**18.3**

Severity by tissue: mean as determined by dividing sum of severity grades for each animal by number of animals in each group (3). Severity grades were 0–4 for normal, minimal, mild, moderate, and marked, respectively.

Total incidence: sum of incidence for each tissue in relation to total tissues scored.

Total severity: sum of mean severity for each tissue.
